# Unraveling Advances in Rice Male Sterility Systems: From Genetic Basis to Hybrid Breeding Innovation

**DOI:** 10.3390/plants15030507

**Published:** 2026-02-06

**Authors:** Wei Liu, Jinlong Ni, Changkai Ma, Jianbo Yang, Shimei Wang, Deze Xu

**Affiliations:** 1 Anhui Province Key Laboratory of Rice Genetics and Breeding, Rice Research Institute, Anhui Academy of Agricultural Sciences, Hefei 230031, China; nauliuwei@163.com (W.L.);; 2 Hubei Province Key Laboratory of Food Crop Germplasm and Genetic Improvement, Food Crops Institute, Hubei Academy of Agricultural Sciences, Wuhan 430064, China

**Keywords:** heterosis, hybrid rice, male sterility, CMS, GMS, molecular mechanisms

## Abstract

Heterosis is a landmark innovation in modern agriculture, which has been widely exploited to boost crop productivity. As a staple food for over half of the global population, rice depends heavily on heterosis for yield improvement. Notably, hybrid rice has made remarkable contributions to global food security over the past several decades. Male sterility serves as the fundamental basis for efficient hybrid rice breeding, with cytoplasmic male sterility (CMS) and genic male sterility (GMS) as the core systems employed in practical production. CMS, induced by mitochondrial genes, can be restored to fertility by nuclear restorer genes, thereby forming the essential genetic basis for the three-line hybrid rice system. GMS, mainly regulated by the nuclear genome, includes dominant and recessive nuclear sterility. Specifically, recessive environment-sensitive genic male sterility (EGMS) has facilitated the development of the two-line hybrid rice system for commercial hybrid seed production. The third-generation hybrid rice technology (TGHRT) is a transgenic approach developed for propagating stable recessive GMS lines. This review comprehensively summarizes the latest advances in rice male sterility systems, focusing on their genetic classification, origin, and molecular mechanisms. It further analyzes their application status, inherent limitations, future research directions, and development trends in hybrid rice production, aiming to deepen our understanding of the innovation and optimization of hybrid rice breeding technologies.

## 1. Introduction

Heterosis refers to the phenomenon whereby the offspring derived from crosses between different varieties or species exhibit superior agronomic traits, such as increased biomass, accelerated development, and enhanced fertility, compared with their parental lines [1,2]. Heterosis has been extensively exploited in agricultural breeding programs to develop crops with improved agronomic performance and desirable characteristics, and is widely considered to be one of the landmark innovations in modern agriculture [2]. Although male sterility is disadvantageous for the reproductive success of individual plants, it serves as a core enabling technology for harnessing heterosis by enabling efficient hybrid breeding systems, thereby substantially enhancing global crop productivity.

Rice (*Oryza sativa* L.) is one of the world’s most important food crops, serving as a stable staple for more than half of the global population. The development of hybrid rice has emerged as a key strategy for boosting yield potential, significantly enhancing food security worldwide over the past several decades, particularly in China. The breeding of hybrid rice varieties relies on male-sterile maternal lines, which fail to produce functional pollen, thus effectively avoiding self-pollination and enabling efficient hybrid seed production [3,4,5]. Several types of male sterility have been applied in rice breeding, including cytoplasmic male sterility (CMS) and genic male sterility (GMS). GMS can be further classified into common nuclear GMS, recessive environment-sensitive genic male sterility (EGMS), and dominant genic male sterility (DGMS). Among these, CMS and EGMS form the basis of the three-line and two-line hybrid rice systems, respectively, which are widely used for commercial hybrid seed production [6]. The third-generation hybrid rice technology (TGHRT) is a transgenic-based approach designed for the propagation and utilization of stable recessive nuclear GMS lines, representing a breakthrough in improving hybrid seed production efficiency [7]. In contrast, DGMS lines are primarily utilized in recurrent selection breeding programs [8]. Specifically, the CMS system, comprising the CMS line, maintainer line, and restorer line, serves as a critical tool for three-line hybrid rice production [9,10,11]. Three-line hybrid rice has been cultivated since the 1970s and is a major type of hybrid rice [3]. In contrast, the two-line breeding systems utilize EGMS lines and restorer lines for hybrid seed production [12,13]. Here, we summarize the latest advancements and achievements in understanding male sterility mechanisms and their practical application in hybrid rice breeding.

## 2. Classification, Origin, and Mechanisms of CMS in Rice

**Figure 1:**
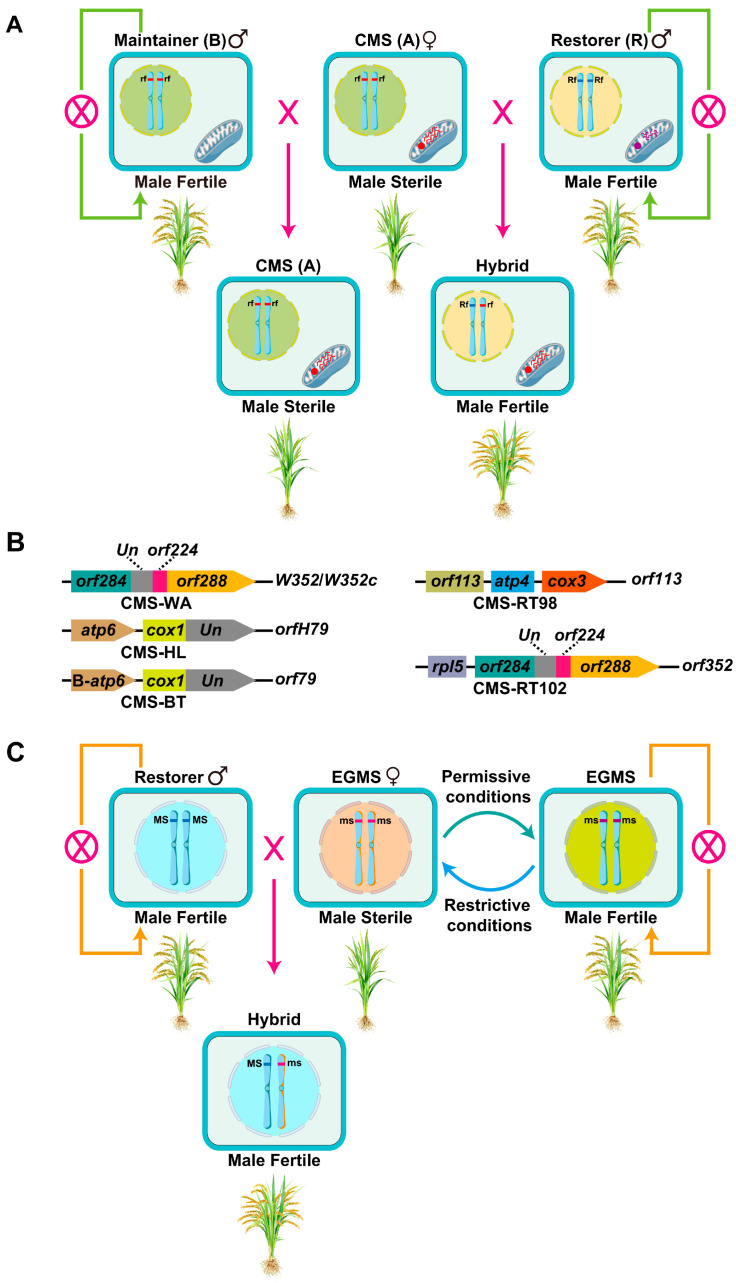
Genetic and mechanistic models of cytoplasmic male sterility (CMS) (**A**) and environment-sensitive genic male sterility (EGMS) (**B**) in rice, respectively. (**A**) The three-line system comprises the CMS line (A line), maintainer line (B line), and restorer line (R line). The CMS line contains a CMS-inducing mitochondrial genome and a nonfunctional restorer of the fertility gene (*Rf*), whereas the maintainer line is isonuclear to the CMS line but possesses a normal mitochondria genome. The restorer line carries a nuclear-encoded *Rf* gene and serves as the male parent for hybrid production. Crosses between the CMS line and maintainer line generate fully male-sterile progeny, which can be used either for hybrid seed production or CMS line propagation. Crosses between the CMS line and restorer line yield fertile F_1_ hybrid seeds. (**B**) Genomic structures of CMS-associated genes in rice. The boxes represent coding sequences, and the horizontal lines indicate flanking regions of the open reading frames (ORFs). The sequences with similarity to the same genes (including flanking and coding sequences) are shown in the same colors. Un, unknown region. (**C**) The two-line system consists of an EGMS line (homozygous for the recessive gene, ms/ms) and a restorer line (homozygous for the dominant wild-type fertility gene, MS/MS). The fertility of the EGMS line is environmentally switchable, enabling hybrid seed production. Specifically, when the EGMS line is male-sterile under restrictive environmental conditions, it functions as the female parent and is crossed with the restorer line to produce F_1_ hybrid seeds. Conversely, under permissive environmental conditions, the EGMS line becomes male-fertile and undergoes self-pollination for line propagation.

CMS is a maternally inherited trait in plants, arising from the dysregulated expression of the mitochondrial and nuclear genomes [14,15]. This impaired cytoplasmic–nuclear crosstalk disrupts the normal development of anther tissues, whether sporophytic or gametophytic, while simultaneously triggering energy metabolism disorders, programmed cell death (PCD) dysregulation, and retrograde signaling imbalance. Collectively, these perturbations result in the formation of non-functional pollen grains [6,15,16]. The three-line system relies on a CMS line, a restorer line, and a maintainer line to produce F_1_ hybrid seeds and maintain the CMS line, respectively (Figure 1A). The CMS line serves as the female parent, harboring male-sterile cytoplasm driven by a CMS gene. In contrast, the maintainer line shares an identical nuclear genome with the CMS line but possesses normal fertile cytoplasm. The seeds of the CMS line are produced by crossing the maintainer line with the CMS line. Both the maintainer and restorer lines are capable of self-pollination to generate their own seeds. Notably, the restorer lines carry one or more functional restorer of fertility (*Rf*) genes and are typically employed as the male parent to hybridize with CMS lines for F_1_ hybrid seeds production [9,10,17]. Based on the sterility mechanisms of CMS and the genetic basis underlying fertility restoration and maintenance, CMS in rice can be divided into three main types: wild abortive CMS (CMS-WA), Boro II CMS (CMS-BT), and Honglian CMS (CMS-HL).

### 2.1. The CMS WA Type

The CMS-WA is derived from common wild rice (*O. sativa* f. *spontanea* L.). In 1970, Yuan Longping’s team discovered a wild rice plant exhibiting pollen abortion in Sanya City, Hainan Province, which was later designated as “Wild abortive” [18]. For the CMS-WA system, pollen sterility occurs at the uninucleate stage. The aborted pollen grains display typical sterile morphological characteristics, classifying CMS-WA as a sporophytic sterility type [18]. Notably, the male sterility of CMS-WA lines maintains high stability across diverse environmental conditions, while their restoration spectrum is restricted to rice species with the AA genome [19]. The first CMS-WA line was successfully developed in 1972. Owing to its prominent advantages, including complete sterility, excellent combining ability, high seed production yield, and strong heterosis in hybrid progeny, CMS-WA lines have been widely applied in hybrid rice production for over three decades, facilitating the development of more than 300 three-line hybrid rice combinations [19,20].

Genetically, CMS-WA is caused by *Wild Abortive 352* (*WA352*/*WA352c*), a novel mitochondrial gene originating from wild rice [9,21]. The *WA352* gene contains three fragments derived from putative mitochondrial ORFs *orf284*, *orf224*, and *orf288*, as well as a short sequence of unknown origin (Figure 1B) [9]. In CMS-WA plants, *WA352* mRNAs are ubiquitously expressed; however, WA352 protein accumulates preferentially in the tapetal cells during the microspore mother cell (MMC) stage and is undetectable in leaf tissues [6]. Further research revealed that WA352 is degraded by the ubiquitin–proteasome system (UPS), and this UPS-mediated regulation restricts WA352 accumulation in anthers, enabling the specific disruption of anther development [22]. Cytochrome c Oxidase (COX11), a nuclear-encoded mitochondrial protein, interacts with WA352. This interaction abolishes the function of COX11 in peroxide metabolism, thereby triggering premature tapetal PCD and ultimately leading to pollen abortion [9,21,22]. *WA314* is another CMS gene found in CMS-WA that causes partial male sterility in transgenic plants [23]. Notably, WA352-induced sterility can be suppressed by two *Rf* genes, *Rf3* or *Rf4*, indicating the existence of distinct nuclear mechanisms to counteract deleterious cytoplasmic factors [9,23,24,25]. Specifically, *Rf4* encodes a pentatricopeptide repeat (PPR) protein with 782 amino acid residues, which suppresses WA352-mediated male sterility by reducing the accumulation of *WA352* mRNA [23,26]. In contrast, *Rf3* is located on rice chromosome 1 but has not yet been cloned. Genetically, it is located between simple sequence repeat (SSR) markers RM443 and RM315, with respective genetic distances of 4.4 cM and 20.7 cM from these markers [27]. *Rf20* is a novel fertility restorer gene specific to CMS-WA, encoding a PPR protein consisting of 440 amino acids residues. *Rf20* exhibits the capacity to restore pollen fertility in some CMS-WA lines under high temperatures. It competes with WA352 to form a complex with COX11, thereby inhibiting reactive oxygen species (ROS) bursts induced by the WA352-COX11 interaction and partially restoring the fertility of sterile lines [28].

### 2.2. The CMS-HL Type

In addition to CMS-WA and CMS-BT, another major type of CMS in rice is CMS-HL. The original CMS-HL line was developed in 1974 by Zhu Yingguo’s team at Wuhan University via backcrossing: *Oryza rufipogon* (a red-awned wild rice accession from Hainan Island) was used as the maternal parent, while Lian-Tang-Zao, an early-maturing *indica* rice variety, served as the recurrent paternal parent [19]. In the CMS-HL system, pollen abortion typically occurs at the binucleate stage, presenting as round abortion. Notably, sterile lines carrying the CMS-HL cytoplasm are classified as gametophytic sterile lines. The genetic relationship between restorers and maintainers of CMS-HL lines differs distinctly from that of CMS-WA lines. Specifically, the Southeast Asian rice variety Pitai exhibits maintainer ability for CMS-HL, whereas early-maturing *indica* rice varieties from the Yangtze River Valley function as restorers for CMS-HL lines [29].

Similarly, the male sterility gene *orfH79* in CMS-HL rice is also located downstream of the mitochondrial *atp6* gene and shares high nucleotide sequence similarity (98%) with *orf79* (Figure 1B). In CMS-HL lines, ORFH79 interacts with the P61 subunit of mitochondrial electron transport chain complex III, which impairs the enzyme activity of mitochondrial complex III. This impairment further induces energy metabolism dysfunction, triggers oxidative stress, and ultimately causes abnormal pollen development [30,31]. Two major *Rf* genes, *Rf5* and *Rf6*, are capable of restoring the fertility of CMS-HL lines [10,32,33,34]. Specifically, *Rf5* is identical to the *Rf1a* gene of CMS-BT, encoding a 791-amino-acid protein that contains 16 PPR motifs. As a core component of the fertility restoration complex, this protein mediates the processing of CMS-associated transcript *atp6-orfH79*, and the complex requires at least two additional members [10,35]. Similarly, *Rf6* encodes a mitochondrial-localized PPR protein of 894 amino acids that contains 21 PPR motifs. The RF6 protein forms a distinct complex with other partner proteins to cleave the aberrant *atp6-orfH79* transcript, thereby restoring fertility [32]. Notably, the coexistence of *Rf5* and *Rf6* can only restore the fertility of 75% of pollen grains in CMS-HL lines [35]. Therefore, to further improve the pollen fertility and seed-setting rate of F_1_ hybrids derived from CMS-HL, the identification and functional characterization of novel *Rf* genes remain an urgent research priority.

### 2.3. The CMS-BT Type

CMS-BT is one of the three major commercially applied CMS types in hybrid rice production. CMS-BT was first developed in 1966 by Japanese researcher Shinjyo from the progenies of the Indian *indica* rice variety Chinsurah Boro II, which served as the female parent, through crossing and subsequent backcrossing with the *japonica* rice variety Taichung 65. In the CMS-BT system, pollen abortion typically occurs at the trinucleate stage. Aborted pollen grains exhibit a spherical morphology and stain blue-black when treated with iodine solution, confirming that CMS-BT is a male sterility type driven by pollen abortion [36]. Notably, consistent with its trinucleate-stage abortion characteristic, CMS-BT is classified as gametophytic male sterility. The genetic relationship between restorers and maintainers of CMS-BT lines is similar to that of CMS-HL lines. Furthermore, both CMS-BT and CMS-HL exhibit a relatively broader fertility restoration spectrum compared with CMS-WA [19].

In the CMS-BT lines, the male sterility gene *orf79*, which encodes a cytotoxic peptide, with its C-terminal region being essential for exerting cytotoxic effects, is located downstream of an extra copy of the *atp6* gene (B-*atp6*) in the mitochondrial genome. Consequently, *orf79* is constitutively co-transcribed with B-*atp6*, generating a chimeric transmembrane protein (Figure 1B). This chimeric protein predominantly accumulates in microspores, ultimately triggering gametophyte abortion in CMS-BT rice [17]. The ORF79 protein accumulates specifically in microspores, and its production is suppressed by the *Rf* genes. Notably, *orf79* expression in both CMS-BT lines and transgenic rice plants induces gametophytic male sterility, which is consistent with the classification of CMS-BT as a gametophytic sterility type [17]. Two fertility restorer genes, *Rf1a* and *Rf1b*, have been identified at the classical *Rf-1* locus, and they are members of a multigene cluster encoding PPR proteins. Both RF1A and RF1B proteins are targeted to mitochondria, where they function to restore male fertility in CMS-BT systems. The *Rf1a* gene promotes the processing of transcripts derived from the CMS-BT-specific mitochondrial operon B-*atp6-orf79* [17,37], whereas *Rf1b* reduces the abundance of dicistronic transcripts of B-*atp6-orf79* [17]. Given that *Rf1a* and *Rf1b* are closely linked on the same chromosome and share highly conserved amino acid sequences, these two *Rf* genes are proposed to be recently duplicated homologous genes. When both restorers are present, RF1A exhibits epistasis over RF1B in the processing of B-*atp6/orf79* mRNA, meaning RF1A’s regulatory effect dominates that of RF1B [17].

### 2.4. Other Types of CMS

In addition to the three major CMS types mentioned above, numerous other CMS types have been reported in rice, including CMS-LD, Dissi CMS (CMS-D), Dian 1 type CMS (CMS-Dian), dwarf wild abortive CMS (CMS-DA), Gambiaka CMS (CMS-G), K52 type CMS (CMS-K), Indonesia 6 (ID) type CMS (CMS-ID), Chinese wild rice type (CMS-CW), CMS-RT98, CMS-RT102, CMS-D1, CMS-TA, and Fujian Abortive CMS (CMS-FA).

CMS-LD was developed in 1971 by Japanese scholar Watanabe through backcrossing the *japonica* cultivar Fujisaka 5 with “Lead Rice” (a rice variety from Burma) [38]. A fertility-restoring gene identified in the *japonica* cultivar Fukuyama was designated *Rf2* [39]. Similar to its role in CMS-BT, the mitochondrial gene *orf79* acts as the CMS-associated gene in CMS-LD. In the presence of *Rf2*, ORF79 accumulation is reduced to nearly zero in CMS-LD and to 25% in CMS-BT, which corresponds to the complete fertility restoration ability of *Rf2* in CMS-LD versus its weak restoration ability in CMS-BT [40]. CMS-D traces its origin to an early-maturing, large-grain line derived from the cross (Dissi D52/37)/Aijiaonante F_7_ [41,42]. Pollen abortion in CMS-D occurs at the uninucleate stage, with aborted pollen exhibiting typical sterile morphology, categorizing it as sporophytic sterility type [41]. CMS-Dian originated from the hybrid progenies of the *japonica* rice variety Taibei 8 and a high-altitude *indica* rice variety. CMS-Dian male sterile lines were subsequently developed by backcrossing with the *japonica* rice “Red Hat Tassel” sterile line. Genetic analyses revealed that the sterility genes and *Rf* genes of CMS-Dian and CMS-BT male sterile lines are allelic, and both systems harbor the same mitochondrial chimeric gene *atp6*-*orf79* [43]. Consequently, CMS-Dian and CMS-BT share identical mechanisms governing pollen sterility and fertility restoration [43]. CMS-DA originated from a male sterile plant discovered in 1970. The CMS-DA sterile line Xieqingzao A was developed via composite hybridization, where the F_1_ generation of (Dwarf Sterile/Junxie) was crossed with Xiezhen 1, and the resulting progeny was subsequently crossed with Xieqingzao. Pollen abortion in CMS-DA occurs at the uninucleate stage, with aborted pollen displaying typical sterile morphology, classifying it as a sporophytic sterility type [44]. CMS-G originated from the progenies of crosses between the African cultivated rice variety Gambiaka and two Chinese rice varieties, Chaoyang 1 and Ya’an. Several CMS-G sterile lines have been developed, including Gang 12A, Chaoyang 1A, Gang 22A, and Ya’an Early A. Pollen abortion in CMS-G occurs at the uninucleate stage, with aborted pollen displaying typical sterile morphology, classifying it as a sporophytic sterility type [45,46]. CMS-K originated from the Yunnan *japonica* rice variety K52. In 1986, the Rice and Sorghum Research Institute of Sichuan Academy of Agricultural Sciences identified the male sterile plants in the F_2_ population of the three-way cross combination “K52/Luhongzao 1//Xinzhennian 2” [47]. For CMS-K, pollen abortion occurs at the uninucleate stage, with aborted pollen exhibiting typical sterile morphological features, placing CMS-K in the category of sporophytic sterility [47]. CMS-ID is derived from the South Asian variety Indonesian Shuitiangu 6. Pollen abortion in CMS-ID occurs at the uninucleate stage, with aborted pollen grains exhibiting typical sterile morphological characteristics, classifying it as a sporophytic sterility type [48]. CMS-CW was developed through backcrossing the *japonica* cultivar Fujisaka 5 with *Oryza rufipogon* L. strain W1. Mature pollen grains of CW-CMS rice exhibit normal morphology under light microscopy and positive fluorochromatic reaction, yet fail to germinate on the stigma [16,49]. *Rf17*, a fertility restorer gene for CMS-CW, has been successfully cloned, and its encoded protein is designated Retrograde-Regulated Male Sterility (RMS). *RMS* encodes a 178-amino acid mitochondrial protein. Notably, RMS mRNA expression in mature anthers is dependent on the cytoplasmic genotype, supporting its candidacy as a retrograde-regulated gene [50]. CMS-RT98 and CMS-RT102 lines were obtained via successive backcrossing between *Oryza rufipogon* W1109, W1125 and *O. sativa* Taichung 65, respectively [51]. For CMS-RT98, the *orf113* gene is co-transcribed with *atp4* and *cox3*, and their transcripts are distinctly processed in the presence of a fertility restorer gene (Figure 1B). The *Rf98* gene for CMS-RT98 encodes a 762-amino acid protein with 18 PPR motifs, which is responsible for the partial restoration of fertility [51]. The *orf352* gene for CMS-RT102 is co-transcribed with the ribosomal protein gene *rpl5*, and the 2.8 kb *rpl5*-*orf352* transcripts are processed into 2.6 kb transcripts with a cleavage inside the *orf352* coding region in the presence of the *Rf* gene (Figure 1B) [52]. CMS-D1 was derived from Dongxiang wild rice in Dongxiang County of Jiangxi Province, China. The CMS-D1 determinant gene *orf182* consists of three recombinant fragments, among which the largest shares high sequence similarity with the mitochondrial genome of *Sorghum bicolor* [53]. The CMS-TA line was generated through successive backcrossing between the *Oryza sativa* cultivars Tadukan and Taichung 65. The *orf312* gene for CMS-TA is similar to the previously described *orf288*, a part of which is among the components comprising *WA352* [54,55]. CMS-FA was developed using the cytoplasm from a common wild rice (*Oryza rufipogon* L.), which was found in Fujian Province, China [11,56,57]. As a sporophytic CMS system, CMS-FA exhibits fertility determined by the parental genotype. Its male sterility is conferred by the chimeric open reading frame *FA182*, a sequence specific to the mitochondrial genome of CMS-FA rice. The restorer gene *OsRf19* encodes a mitochondrion-targeted PPR protein, which mediates the cleavage of *FA182* transcripts to restore male fertility [58].

## 3. Classification, Origin, and Mechanisms of EGMS in Rice

The two-line hybrid rice system comprises an EGMS line carrying a recessive EGMS gene and a paternal line with a distinct nuclear genome [59,60]. As a type of nuclear gene-controlled male sterility, EGMS is defined by a reversible switch in male fertility in response to specific environmental stimuli [61]. Under permissive environmental conditions, EGMS are male fertile and capable of self-pollination to maintain the EGMS line itself. In contrast, under restrictive conditions, they revert to male sterile, enabling crossing with most conventional rice varieties to produce hybrids with heterotic performance (Figure 1C). In rice, EGMS can be classified into four main types based on their responsiveness to photoperiod, temperature, nitrogen status, or humidity in rice [62]. Generally, photoperiod-sensitive genic male sterility (PGMS) typically exhibits male sterility under long-day (LD) conditions and male fertility under short-day (SD) conditions. Conversely, “reverse PGMS” displays male sterility under SD conditions and male fertility under LD conditions. Thermosensitive genic male sterility (TGMS) generally shows male sterility at high temperatures and fertility at low temperatures, while “reverse TGMS” exhibits the inverse phenotype: male sterility at low temperatures and male fertility at high temperatures [62]. Humidity-sensitive genic male sterility (HGMS) is characterized by male sterility under low-humidity conditions and fertility under high-humidity conditions [63]. Additionally, nitrogen-sensitive genic male sterility (NGMS) is defined by male sterility under nitrogen-deficient conditions and fertility under nitrogen-sufficient conditions [64].

In recent years, studies of EGMS genes in rice have achieved remarkable progress; numerous key EGMS genes have been cloned and the molecular mechanisms by which these genes regulate the EGMS trait have been gradually elucidated. To date, a number of EGMS-related genes have been mapped in rice, among which 19 have been successfully cloned and functionally characterized [61]. A deeper understanding of EGMS system in rice is of great significance for advancing two-line hybrid rice breeding, especially in the context of increasingly variable and unpredictable global climates.

### 3.1. Long Noncoding RNAs Regulate P/TGMS

**Figure 2:**
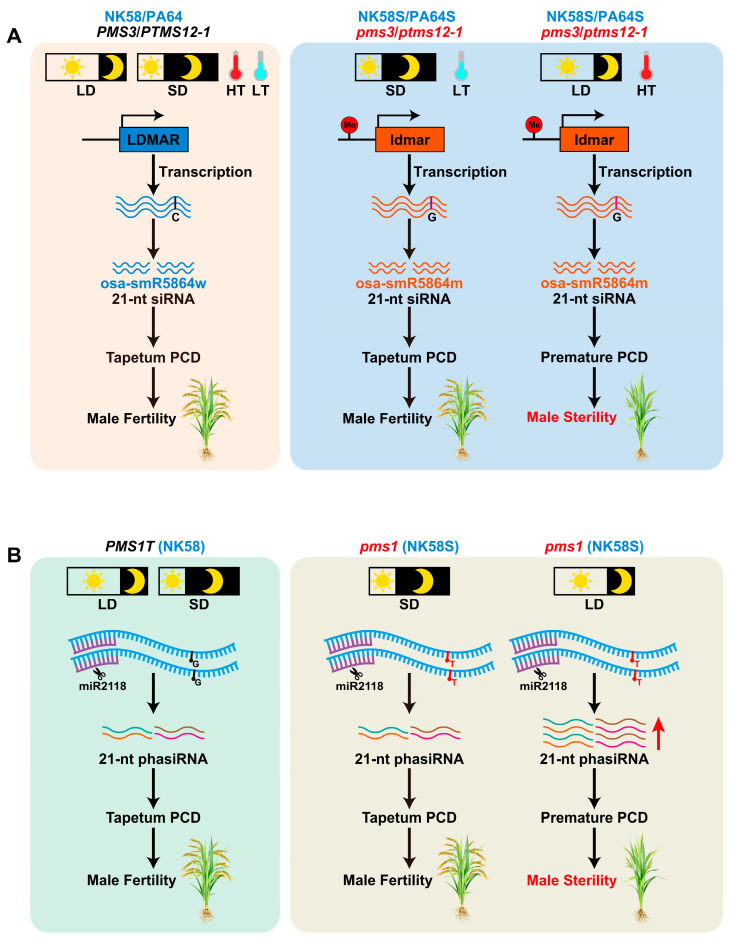
Molecular mechanisms of *pms3* (also known as *p/tms12-1*) (**A**) and *pms1* (**B**) in regulating photoperiod-sensitive genic male sterility (PGMS) in rice, respectively. (**A**) *PMS3* (or *P/TMS12-1*) encodes a long noncoding RNA (lncRNA) that modulates PGMS in NK58S (or PA64S). *PMS3* encodes a long-day-specific male-fertility-associated RNA (LDMAR). A G-to-C SNP within LDMAR is proposed to alter its secondary structure, which in turn induces elevated DNA methylation in the putative promoter region of LDMAR. This methylation event specifically represses LDMAR transcription under long-day conditions, triggering PCD in developing anthers and ultimately leading to PGMS in NK58S. Alternatively, *P/TMS12-1* encodes an lncRNA that generates a 21-nt small RNA called osa-smR5864w. The G-to-C mutation in this small RNA yields the variant osasmR5864m, which loses its target regulatory function on distinct genes in *japonica* (NK58S) and *indica* (PA64S) genetic backgrounds, ultimately resulting in PGMS and thermosensitive genic male sterility (TGMS), respectively. (**B**) The *pms1* locus also encodes an lncRNA *PMS1T* that regulates PGMS in NK58S. The *PMS1T* transcript is targeted by miR2118, triggering the biogenesis of 21-nt phased small interfering RNAs (phasiRNAs). A G-to-T mutation adjacent to the miR2118 cleavage site may alter the RNA secondary structure of PMS1T, thereby modulating the efficiency of miR2118-mediated cleavage in NK58S. Enhanced cleavage efficiency promotes increased phasiRNA production, and these excess phasiRNAs are hypothesized to target downstream genes involved in anther development, ultimately resulting in male sterility in NK58S under long-day conditions. LD, long-day conditions; SD, short-day conditions; HT, high temperatures; LT, low temperatures.

RNA processing has been demonstrated to play a critical role in regulating PGMS in rice. Specifically, two long noncoding RNAs (lncRNAs) that are processed into 21-nt phased small interfering RNAs (phasiRNAs) have been shown to be involved in regulating the PGMS trait in rice [12,65]. The *photoperiod-sensitive male sterility 3* (*pms3*) gene was previously identified as the causal locus underlying the conversion of the fertile rice line Nongkeng58 (NK58) to the PGMS line NK58S [66]. Fine mapping and comparative sequencing analyses revealed a single-nucleotide polymorphism (SNP) resulting from G-to-C substitution at the *pms3* locus in NK58S relative to NK58 [67]. Notably, this SNP is located within a lncRNA, and functional validation showed that overexpression of the lncRNA fragment from NK58 in NK58S restored fertility under LD conditions. Subsequently, a 1236-base lncRNA, designated long-day-specific male-fertility-associated RNA (LDMAR), was identified as the candidate gene corresponding to the pms3 locus. LDMAR is expressed in almost all rice tissues, with relatively higher expression in young panicles, and its expression level is significantly higher in NK58 than NK58S under LD conditions. It has been hypothesized that sufficient accumulation of LDMAR transcripts is essential for normal pollen development under LD conditions. The G-to-C SNP is thought to alter the secondary structure of LDMAR, which in turn induces increased methylation in the putative promoter region of LDMAR. This methylation specifically reduces LDMAR transcription under LD conditions, triggering PCD in developing anthers and ultimately leading to PGMS (Figure 2A) [12].

Further research revealed that the promoter region of LDMAR contains a small interfering RNA (siRNA) designated Psi-LDMAR, whose abundance is significantly higher in NK58S than in NK58. Studies suggest that Psi-LDMAR may originate from the sense transcript of AK111270, a transcript whose 3′ end overlaps with the 5′ end of LDMAR by 110 bp. Overexpression of AK111270 in NK58S led to an increase in Psi-LDMAR levels, which in turn induced RNA-directed DNA methylation (RdDM) in the LDMAR promoter. This RdDM-mediated hypermethylation elevated the methylation level of the LDMAR promoter, suppressed LDMAR transcription, and ultimately resulted in male sterility in NK58S [68].

Another research group reported that the *photo- or thermo-sensitive genic male sterility locus on chromosome 12* (*p/tms12-1*) confers PGMS in the *japonica* rice line NK58S and TGMS in the *indica* rice line Peiai 64S (PA64S, derived from NK58S). Notably, *p/tms12-1* is identical to *pms3*, the locus previously shown to confer PGMS in the NK58 genetic background. Consistent with the *PMS3* candidate gene LDMAR, *P/TMS12-1* also encodes an lncRNA and produces a 21-nt small RNA called osa-smR5864w. Intriguingly, the expression level of *P/TMS12-1* itself is not affected by variations in temperature or day length. Functional analysis revealed that osa-smR5864w suppresses the expression of its target genes, while a mutation in this small RNA resulting in the variant osa-smR5864m, and leads to loss of targeting function for different genes in *japonica* (NK58S) versus *indica* (PA64S) genetic backgrounds, ultimately resulting in PGMS and TGMS, respectively (Figure 2A) [13].

Another PGMS gene, *photoperiod-sensitive male sterility 1* (*pms1*), is a semi-dominant allele that encodes an lncRNA named *PMS1T*. This lncRNA is targeted by a 22-nt miR2118, a microRNA preferentially expressed in the immature inflorescences and known to trigger the biogenesis of 21-nt phasiRNAs in plants. Small RNA sequencing of young panicles revealed that 18 pairs of 21-nt phasiRNAs are generated from the *PMS1T* transcript. Comparative analysis further showed that the abundance of 21-nt *PMS1T*-derived phasiRNAs is higher in NK58S under LD conditions at the MMC stage than under SD conditions. A G-to-T mutation was identified in one of these phasiRNA, located 24 nt downstream of the miR2118-directed cleavage site. This SNP is hypothesized to alter the RNA secondary structure of *PMS1T*, thereby modifying the efficiency of miR2118-mediated cleavage. Enhanced cleavage efficiency would lead to increased phasiRNA production, and these excess phasiRNAs may then target downstream anther development-related genes, ultimately resulting in male sterility in NK58S (Figure 2B). Sufficient accumulation of the LDMAR transcript is required for fertility under LD conditions, whereas abundance of *PMS1T*-phasiRNAs is the main cause for fertility reduction [65]. Collectively, these findings highlight the critical role of the noncoding RNAs in regulating P/TGMS in rice. However, despite the cloning and characterization of these genes and their molecular mechanisms, how they govern the development of MMCs and tapetal cells, as well as the potential genetic interactions between them, remain to be elucidated.

Heading date (or flowering time) determines the seasonal and regional adaptation of rice varieties and is mainly controlled by photoperiod sensitivity, which is a typical quantitative trait regulated by multiple genes [69]. Previous studies have identified two major signaling pathways regulating flowering in rice. The first is the evolutionarily conserved photoperiodic flowering pathway, *OsGI*-*Hd1*-*Hd3a*/*RFT1*, which is analogous to the *Arabidopsis GI*-*CO*-*FT* pathway. In addition to this conserved pathway, rice possesses another monocot-specific pathway, in which *Ehd1* acts as a central hub and is regulated by multiple flowering genes, including *Ghd7*, *MADS50*, *Ehd2*, *Ehd3*, and *Ehd4* [69,70]. *Early heading date 1* (*Ehd1*) integrates various upstream signals and promotes the expression of the downstream florigen genes *Heading date 3a* (*Hd3a*) and *RICE FLOWERING LOCUS T1* (*RFT1*) under both LD and SD conditions [69,71]. Among the upstream regulators of *Ehd1*, *Heading date 1* (*Hd1*), *Grain number*, *plant height*, *and heading date 7* (*Ghd7*), *Days to heading on chromosome8* (*DTH8*), and *PSEUDO-RESPONSE REGULATOR37* (*PRR37*) are the core genes in the regulation of rice photoperiod sensitivity [4,69,72,73,74]. Recently, a CCHC-type zinc finger protein, ELD1, was found to be essential for rice embryo survival. Notably, partial loss of ELD1 function results in early flowering under LD conditions [75]. Previous results showed that *Hd1* alone promotes heading regardless of day length. However, under LDs, *Hd1* collaborates with functional Ghd7 or *Ghd7*/*DTH8* to suppress flowering by negatively regulating the *Ehd1*-*Hd3a*/*RFT1* pathway, thereby delaying heading [74]. Specifically, under LD conditions, rice lines harboring only *Hd1* but lacking *Ghd7*/*DTH8* exhibited reduced plant height, fewer panicle branches, and mild sterility, the latter arising from incomplete anther development induced by premature heading. Conversely, lines carrying *Ghd7/DTH8* (with non-functional *hd1*) display delayed heading, enhanced fertility, and improved agronomic performance [74]. Beyond its canonical role as a molecular switch for heading, *Ehd1*-*Hd3a*/*RFT1* module also regulates anther wall formation, microspore meiosis, and pollen germination in an expression-level-dependent manner. Conversely, the synergistic inhibition of the *Ehd1-Hd3a/RFT1* module by *Hd1/Ghd7/DTH8* not only delays heading but also concomitantly increases the risk of pollen abortion [74].

### 3.2. Transcriptional Regulation Controlling P/TGMS

Transcription factors (TFs) play a pivotal role in mediating anther and pollen development in response to environmental cues [76,77]. Two MYB family TFs have been identified as key regulators of PGMS in rice. The *carbon starved anther* (*csa*) mutant, a rice reverse PGMS line, exhibits male sterility under SD conditions and male fertility under LD conditions [78,79]. In contrast, the *csa2* mutant shows partial sterility under extended daylight conditions and complete fertility under SD conditions. Notably, the *csa csa2* double mutant displays semi-sterility (similar to *csa2*) under LD conditions but complete sterility (similar to *csa*) under SD conditions [80]. These results suggest that *CSA* and *CSA2* are not functionally redundant and play distinct roles in regulating anther development under SD and LD conditions, respectively.

**Figure 3:**
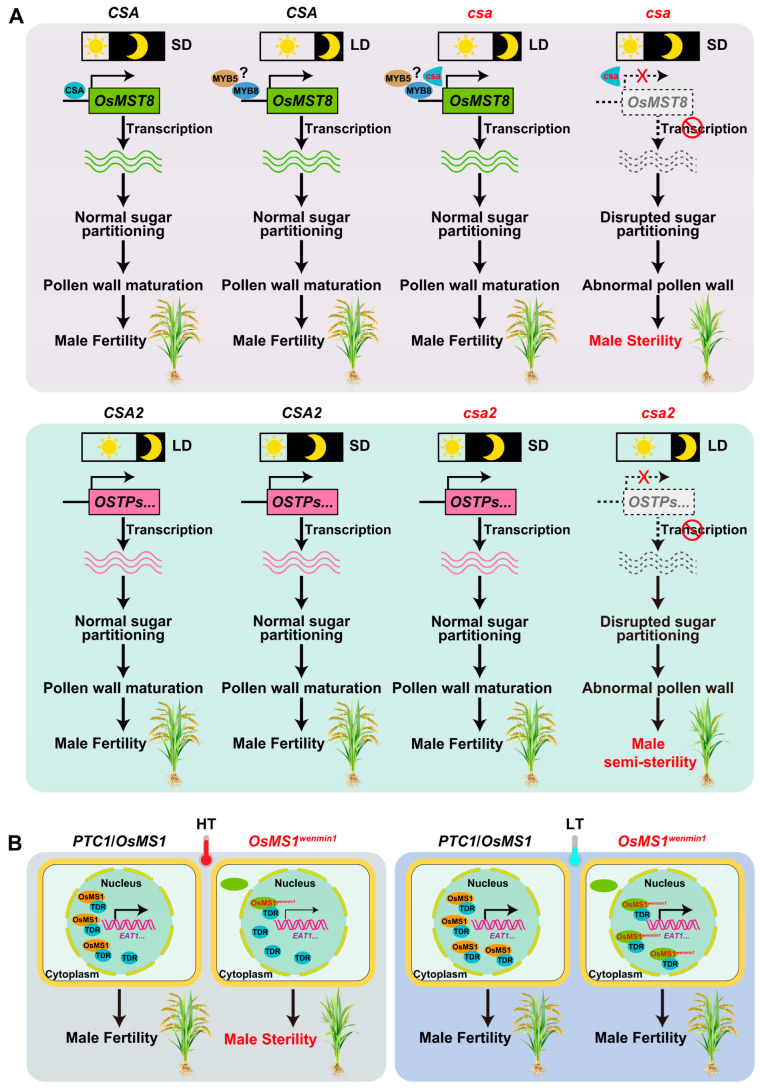
Mechanism of *CSA*, *CSA2* (**A**), and *OsMS1^wenmin1^* (**B**) in regulating PGMS and TGMS in rice, respectively. (**A**) *Carbon Starved Anther* (*CSA*) and its homologous *CSA2* are key regulators of sugar partitioning during male reproductive development. CSA directly binds the promoter of the monosaccharide transporter *OsMST8* to modulate sugar transportation. Under short-day conditions, the *csa* mutant exhibits reduced anther sink activity via downregulated *OsMST8*, impairing anther sugar accumulation and causing male sterility. Under long-day conditions, other regulatory factors (e.g., MYB5 and MYB8) may compensate for CSA function to ensure proper pollen wall formation. *CSA2* is hypothesized to mediate fertility transition by regulating the transcription of other sugar transporter proteins (OSTPs). Specifically, the *csa2* mutant exhibits male semi-fertility under long-day conditions due to insufficient OSTP-mediated sucrose transport in anthers, but maintains male fertility under short-day conditions. (**B**) Molecular pathway of TGMS regulated by *OsMS1^wenmin1^*. The wild-type OsMS1 protein is primarily localized in the nucleus, whereas the mutant OsMS1^wenmin1^ protein is localized in both nucleus and cytoplasm. OsMS1 physically interacts with TDR to bind the promoter of *EAT1*. At high temperatures, the abundance of OsMS1 proteins is decreased, but there are still enough OsMS1 proteins in the nucleus to interact with TDR to activate the expression of *EAT1*, thus retaining pollen fertility. In contrast, the insufficient nuclear-localized OsMS1^wenmin1^ fails to interact with TDR, leading to a dramatic reduction in downstream gene expression and the formation of sterile pollen. At low temperatures, adequate nuclear protein levels of OsMS1 and OsMS1^wenmin1^ support normal pollen development and male fertility restoration. LD, long-day conditions; SD, short-day conditions; HT, high temperatures; LT, low temperatures.

Molecular characterization revealed that *CSA* encodes an R2R3 MYB TF, with its expression primarily detected in vascular tissues and the tapetum, while *CSA2* is specifically expressed in anthers [79,80]. This differential expression pattern suggests that the biological functional divergence between *CSA* and *CSA2* [80]. Mechanistically, *CSA* directly regulates the expression of the monosaccharide transporter gene *Monosaccharide Transporter 8* (*OsMST8*) [78,79]. Mutation of *CSA* significantly reduces *OsMST8* expression, impairing sugar translocation from the flag leaf to the lemma/palea and anther via the stem. This disruption in sugar partitioning ultimately leads to male sterility in the *csa* mutant under SD conditions (Figure 3A) [79].

Consistent with *CSA*, *CSA2* also encodes a MYB TF but does not directly regulate *OsMST8*, suggesting it may target other carbohydrate transport-related genes. Notably, driving *CSA2* expression via the *CSA* promoter restored male fertility in the *csa* mutant under SD conditions, indicating potential overlap in their molecular functions. Under LD conditions, the *csa2* mutant fails to efficiently translocate sugars from flag leaves to anthers, resulting in semi-male sterility. Collectively, these findings indicate that *CSA* and *CSA2* share a common core molecular function, regulating sugar transportation from leaves to anthers in response to photoperiod to support pollen development, while their differential expression and target specificity enable context-dependent regulation of PGMS (Figure 3A) [80].

*PERSISTENT TAPETAL CELL1* (*PTC1*)/*Male Sterility 1* (*OsMS1*)/*tms9-1* encodes a PHD-finger TF that regulates pollen exine formation and tapetum PCD in rice [81,82,83,84]. *PTC1* is expressed specifically in tapetal cells and microspores during anther development at stages 8 and 9. The *ptc1* mutant displays a previously unreported phenotype of uncontrolled tapetal proliferation followed by necrosis-like tapetal death [81]. To further investigate the mechanism underlying this gene’s regulation of PCD in rice, a homozygous mutant named *osms1* was generated using the CRISPR/Cas9 gene-editing system. The *osms1* mutant displayed complete male sterility with slightly yellow and small anthers, as well as invisible pollen grains [83]. Additionally, cytological observation and TUNEL assays revealed delayed tapetal PCD, defective pollen exine formation, and absence of DNA fragmentation in the anthers of the *osms1* mutant. Subcellular localization analysis showed that OsMS1 localizes to the nucleus of rice protoplasts. Yeast two-hybrid (Y2H) and bimolecular fluorescence complementation (BiFC) assays demonstrated that OsMS1 interacts with OsMADS15 and TDR INTERACTING PROTEIN2 (TIP2). Previous studies have reported that TIP2 coordinates with TDR to modulate EAT1 expression, thereby regulating tapetal PCD in rice. Collectively, these results indicate that OsMS1 regulates tapetal PCD and pollen exine formation in rice by interacting with known tapetal regulatory factors via its PHD finger [83]. The natural allele *OsMS1^wenmin1^* confers TGMS in rice [84]. Sequence analysis revealed that *OsMS1* containing a putative nuclear localization signal (NLS) at the N-terminus and an LXXLL motif in the middle region. A T-to-C substitution within the LXXLL motif of the OsMS1^wenmin1^ results in a Leu-to-Pro amino acid change, which confers the TGMS trait. *OsMS1^wenmin1^* regulates TGMS through temperature-dependent transcriptional control. The wild-type OsMS1 protein is primarily localized in the nucleus, whereas the mutant OsMS1^wenmin1^ protein is localized in both the nucleus and cytoplasm, indicating that the mutation in OsMS1^wenmin1^ disrupts its subcellular localization. Further studies showed that temperature modulates the abundances of both OsMS1 and OsMS1^wenmin1^ proteins, with OsMS1^wenmin1^ exhibiting greater sensitivity to temperature fluctuations. At restrictive temperatures, the nuclear abundance of OsMS1^wenmin1^ is significantly lower than that of OsMS1. OsMS1 can interact with the tapetal PCD regulator Tapetum Degeneration Retardation (TDR) to bind the promoter of its downstream target gene *Eternal Tapetum 1* (*EAT1*) in a temperature-dependent manner. At permissive temperatures, both the wild-type *OsMS1* and *OsMS1^wenmin1^* allele maintain normal pollen development via appropriate protein levels, respectively. Both proteins interact with the TDR to activate the expression of downstream genes, ultimately producing fertile pollen. At restrictive temperatures, the abundance of OsMS1 proteins was decreased, but sufficient nuclear OsMS1 remains to interact with TDR to activate the expression of downstream genes, thus retaining pollen fertility. In contrast, high temperatures more drastically reduce the level of OsMS1^wenmin1^ proteins. As a result, insufficient nuclear OsMS1^wenmin1^ fails to interact with TDR, leading to a dramatic reduction in downstream gene expression and the formation of sterile pollen (Figure 3B). Collectively, OsMS1 mediates fertility–sterility conversion in response to temperature fluctuations by modulating its nuclear localization and abundance [84].

### 3.3. Slow Development Contributes to Fertility Restoration in TGMS Rice

Recent studies have demonstrated that slow development is a conserved mechanism underlying fertility restoration in P/TGMS lines in *Arabidopsis* [85,86]. This theory posits that permissive environmental conditions, including low temperature, low light intensity, and SD photoperiod, can retard anther development, thereby enabling the formation of functional pollens in P/TGMS lines [85,86,87,88,89,90].

Reproductive processes, including tapetum initiation, sporopollenin synthesis, and pollen wall formation are conserved between *Arabidopsis* and other crops. Consistent with the *Arabidopsis* model, slow pollen development has also been observed in rice under low-temperature conditions [86], and multiple rice TGMS mutants rely on this mechanism for fertility restoration [91,92,93]. A well-characterized example is the *OsTMS18*/*No Pollen 1* (*OsNP1*) gene, which encodes a glucosemethanol-choline oxidoreductase. A point mutation (Gly-to-Ser substitution) in *OsTMS18* gives rise to the TGMS mutant *ostms18*, which exhibits male sterility with pollen abortion at high temperatures. At low temperatures, however, slow anther development reduces the biological demand for robust cell wall protection, and the flawed pollen wall in *ostms18* is sufficient to shield microspores, enabling the formation of functional pollen and thus restoring fertility [93]. Another TGMS-related gene, *OsTMS15*, encodes a leucine-rich repeat receptor-like kinase (LRR-RLK) protein multiple sporocyte 1 (MSP1). MSP1 is known to interact with its ligand Tapetum Determinant1-Like (OsTDL1A) to initiate tapetum development, a process essential for pollen formation. In the *ostms15* mutant, a point mutation from GTA (Val) to GAA (Glu) in the TIR motif of the LRR region reduces its interaction with OsTDL1A, thereby conferring the TGMS phenotype. Notably, the CRISPR/Cas9-generated knockout mutant *ostms15-cr* exhibits complete sterility at both high and low temperatures. In contrast, the weak allelic mutant *ostms15* exhibits compromised tapetum function at high temperatures, and this defect can be compensated for by enhanced interaction with OsTDL1A and slow development at low temperatures, which restores functional tapetum and thus fertility [91]. The TGMS mutant *ostms16* exhibits abnormal pollen exine at high temperatures but regains fertility at low temperatures. *OsTMS16* encodes a fatty acyl-CoA reductase (FAR), and a single-base mutation in this gene reduces its enzymatic activity, resulting in defective pollen walls. At high temperatures, the mutant protein mOsTMS16^M549I^ fails to provide sufficient protection for microspores, leading to sterility. At low temperatures, however, the enzymatic activity of mOsTMS16^M549I^ is closer to that of wild-type OsTMS16, allowing the imperfect exine to still support microspore maturation [92]. Notably, all these TGMS genes are tightly linked to core processes of anther development and pollen formation. Generally, plants typically exhibit accelerated growth under moderately high temperatures and long photoperiods, whereas growth is slowed under relatively low temperatures and short photoperiods. Collectively, these studies confirm that slow anther development is a common, conserved mechanism that restores the fertility under permissive conditions in TGMS rice.

### 3.4. ROS Homeostasis Regulating P/TGMS

ROS homeostasis is critical for plant reproductive development. During male gametophyte development, ROS levels are dynamically and precisely regulated, and this balance is essential for mediating tapetal degradation, microspore development, and pollen maturation [92,94]. Specifically, ROS begin to accumulate during the initial phase of meiosis, increase progressively throughout the meiotic stage, and reach a peak during the late stage of anther development, reflecting their spatiotemporally controlled role in reproductive processes [92].

The mutant *ostms19*, which exhibits sterility at high temperatures and fertility at low temperatures, exemplifies the link between ROS homeostasis and fertility conversion. *OsTMS19* encodes a novel PPR protein that is essential for pollen formation. A point mutation (GTA encoding Val to GCA encoding Ala) in *OsTMS19* confers the P/TGMS phenotype in *ostms19*. Expression and subcellular localization analyses show that *OsTMS19* is highly expressed in the tapetum and localized to mitochondria, consistent with the role of mitochondria in ROS production and scavenging. Under high-temperature or LD photoperiod conditions, the pollen nuclei of *ostms19* exhibit significantly higher DNA damage, accompanied by excessive ROS accumulation in anthers during pollen mitosis. This ROS overaccumulation disrupts the expression of genes involved in pollen development and impairs pollen intine formation, ultimately leading to male sterility. In contrast, under low-temperature or SD photoperiod conditions, ROS in *ostms19* anthers can be effectively scavenged, restoring ROS homeostasis and enabling normal pollen development, thereby recovering fertility. These findings underscore that maintaining ROS homeostasis is pivotal for fertility restoration in *ostms19* [95]. Notably, this regulatory relationship between ROS homeostasis and fertility conversion is not unique to *ostms19*; it has also been observed in other tested rice P/TGMS lines. Beyond elucidating a conserved mechanism for P/TGMS fertility control, these findings provide valuable insights into how sporophytic genes can influence gametophytic processes, highlighting the interconnectedness of sporophytic and gametophytic regulation in male reproductive development.

### 3.5. RNA Processing Regulating TGMS

Temperature-dependent mRNA splicing has been identified as a key regulatory mechanism contributing to TGMS in rice [62]. A well-characterized example of this mechanism in the regulation of *Ugp1*, which encodes UDP-glucose pyrophosphorylase (UGPase), an enzyme that catalyzes the reversible conversion of glucose-1-phosphate and UTP to UDP-glucose and pyrophosphate [96]. *Ugp1* is specifically expressed during anther development and catalyzes the production of UDP-glucose and pyrophosphate. Functional studies confirm that the *Ugp1*-silenced or -overexpression rice plants are male sterile. Specifically, *Ugp1* silencing via RNAi or co-suppression disrupts normal pollen development, but only co-suppression lines display temperature-dependent sterility.

**Figure 4:**
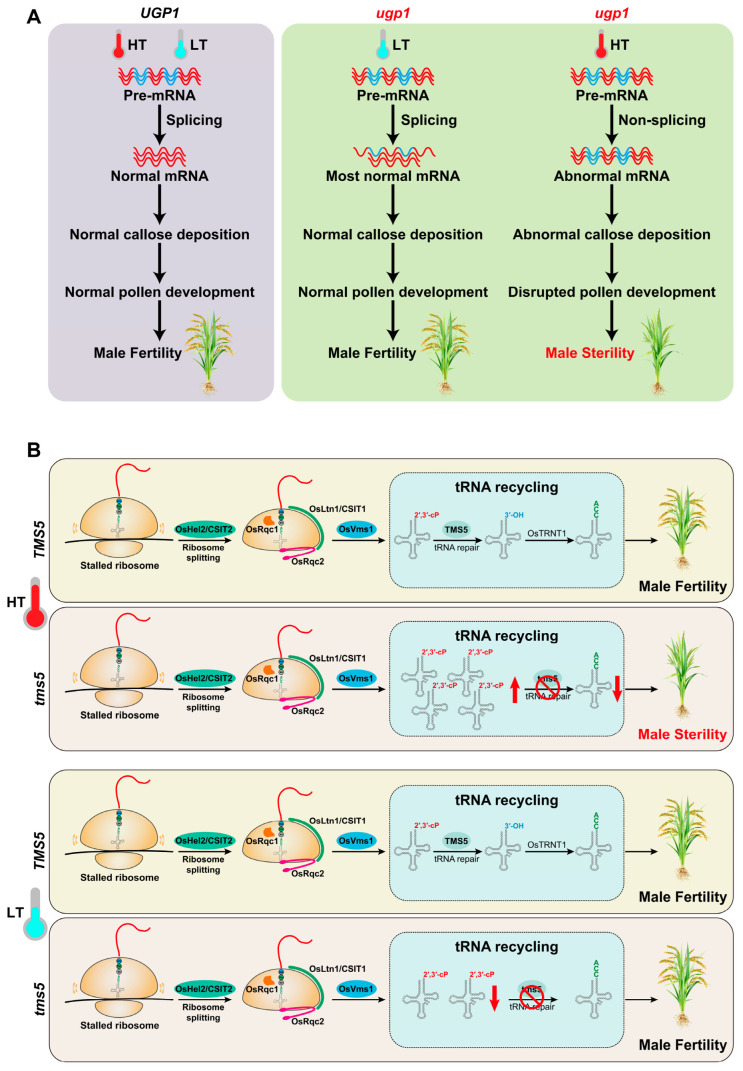
Mechanism of *Ugp1* (**A**) and *TMS5* (**B**) in regulating TGMS in rice, respectively. (**A**) In *Ugp1*-overexpressing lines, the intron of *Ubi1* promoter fails to be accurately spliced from the *Ugp1* transcript at high temperatures, generating aberrant mRNA molecules that are incompetent for translating functional UGPase, ultimately resulting in male sterility. Conversely, at low temperatures, a subset of the intron-containing aberrant *Ugp1* transcripts undergo correct splicing, restoring the biosynthesis of functional UGPase and thereby recovering male fertility. HT, high temperatures; LT, low temperatures. (**B**) A working model elucidating impaired cyclic phosphate (cp) tRNA repair underlying tms5-mediated TGMS in rice. At high temperatures, in the presence of more ribosome stalling events, the E3 ligase OsHel2/CSIT2 could sense stalled ribosomes and promote their dissociation into its 60S and 40S subunits. Subsequently, OsRqc2, OsLtn1/CSIT1, and OsRqc1 associate with the talled 60S ribosomal subunits to form nascent chain-tRNA/60S complexes. The endonuclease OsVms1 specifically cleaves off the 3′CCA of 60S-associated peptidyl-tRNAs to generate 2′,3′-cP on the 3′ end of the tRNA. Wild-type TMS5 could remove 2′,3′-cP on OsVms1-cleaved ΔCCA-tRNAs which allows tRNA nucleotidyl transferase 1 (TRNT1) to add the CCA for tRNA recycling, thus replenish the pool of mature tRNAs and thereby sustaining male fertility. In the *tms5* mutant, the cP-ΔCCAtRNA repair activity is impaired, cP-ΔCCA-tRNAs over-accumulated and cause a reduction in the pool of mature tRNAs, ultimately leading to pollen abortion. At low temperatures, the production of cP-ΔCCA-tRNAs is negligible, and the supply of mature tRNAs remains sufficient, making the *tms5* mutant fertile. HT, high temperatures; LT, low temperatures.

In *Ugp1* RNAi plants, gene silencing leads to a drastic reduction in UGPase activity, impairing callose deposition during pollen cell development. This callose deficiency triggers the degradation of MMCs in the early meiotic stage, ultimately resulting in complete and stable pollen abortion. In contrast, *Ugp1* co-suppression plants exhibit unstable, temperature-dependent male sterility: they are sterile at high temperatures but regain fertility at low temperatures. The temperature sensitivity of co-suppression lines originates from temperature-dependent splicing of *Ugp1* transcripts. In *Ugp1*-overexpressing lines, the intron of *Ubi1* fails to be accurately spliced from the *Ugp1* transcript, generating aberrant mRNA molecules that cannot be translated into functional UGPase at high temperatures. The complete loss of UGPase activity at high temperatures recapitulates the sterility phenotype observed in RNAi lines. Conversely, at low temperatures, a subset of the aberrant *Ugp1*-*Ubi1* intron transcripts undergoes correct splicing, restoring the production of functional UGPase. The resulting UGPase protein level in co-suppression lines grown at low temperatures is equivalent to that in wild-type plants, which rescues callose deposition, MMC survival, and pollen development, thereby restoring fertility (Figure 4A) [96]. This study highlights temperature-dependent mRNA splicing as a distinct regulatory layer governing TGMS in rice, where environmental temperature modulates transcript processing efficiency to control the expression of a key metabolic enzyme, ultimately mediating fertility conversion.

### 3.6. Signal Transduction Processes Regulating TGMS

RLKs are a large family of membrane-localized proteins that play pivotal roles in regulating diverse biological processes in plants, including male reproductive development, pathogen resistance, and PCD [97,98,99]. A previous study has identified two LRR-RLKs, Thermo-Sensitive Genic Male Sterile 10 (TMS10) and its close homolog TMS10-Like (TMS10L), which redundantly regulate tapetum degeneration and pollen development in response to temperature fluctuations, thereby mediating TGMS in rice [100].

The kinase activity of TMS10 is critical for maintaining pollen viability at relatively higher temperatures. Loss-of-function mutations in *TMS10* induce male sterility at high temperatures, whereas male fertility is restored at low temperatures in rice. In contrast, disruption of *TMS10L* exerts no obvious effect on fertility, with the mutant plants remaining fertile at both high- and low-temperature conditions. Notably, the *tms10 tms10l* double mutant exhibits complete male sterility regardless of temperature regimes. This phenotypic discrepancy between single and double mutants clearly demonstrates that *TMS10* and *TMS10L* exhibit functional redundancy in modulating pollen development. Specifically, *TMS10L* can compensate for the loss of *TMS10* function at low temperatures, whereas this compensatory capacity is insufficient at high temperatures. Furthermore, expression profiling analyses showed that the transcript level of *TMS10L* at low temperatures was three- to five-fold higher than that at high temperatures, indicating that *TMS10L* is transcriptionally upregulated in response to low temperatures and thus plays a more prominent role in anther development at low temperatures. This temperature-dependent upregulation of *TMS10L* explains its ability to complement *TMS10* function in the *tms10* single mutant, thereby restoring pollen fertility at low temperatures. Additionally, compared with the wild-type plants, the mRNA level of *TMS10L* was significantly reduced in *tms10* anthers at both high and low temperatures, suggesting that *TMS10* not only acts as a temperature-responsive kinase but also modulates the expression of its homolog *TMS10L*. Collectively, these findings demonstrate that TMS10 and TMS10L function as a key molecular switch in postmeiotic tapetal development and pollen maturation by buffering environmental temperature variations [100].

### 3.7. Ribosome-Associated Protein Quality Control (RQC) Regulating TGMS

The RQC pathway is well characterized for its roles in rescuing stalled ribosomes, recycling transfer RNAs (tRNAs), and degrading abnormal nascent polypeptide chains, thereby maintaining protein homeostasis [101,102]. Beyond protein homeostasis, RQC systems also play vital roles in regulating TGMS, as evidenced by the functional characterization of the TGMS gene *Thermo-Sensitive Genic Male Sterile 5* (*TMS5*) and its interacting RQC components in rice [103,104,105,106,107,108].

Recent studies have further elucidated the mechanisms underlying *tms5* mutants and revealed that TMS5 acts as a tRNA cyclic phosphate (cP)-specific repair enzyme, which is essential for repairing cP-ΔCCA-tRNA and maintaining fertility at high temperatures. Loss of TMS5 function leads to the overaccumulation of cP-ΔCCA-tRNA and insufficiency of mature tRNAs at high temperatures. Notably, knocking out *OsVms1*, an enzyme that generates cP-ΔCCA-tRNA, completely restored fertility in *tms5* mutants at high temperatures. This finding demonstrated that the *TMS5* mutation impairs cP-tRNA repair and disrupts tRNA recycling, ultimately resulting in TGMS [106]. Our recent study found that the E3 ligase OsHel2 cooperates with TMS5 to regulate rice fertility by impeding readthrough of stalled mRNAs in the RQC pathway at restrictive temperatures. The point mutation (C29S) in the RING domain of OsHel2 blocked its E3 ligase activity and resulted in partial readthrough of the stalled sequences, thereby enabling escape from RQC pathway surveillance in the suppressor line *tms5 oshel2-1*. Only a small portion of stalled ribosomes entered the tRNA recycling process. Consequently, the levels of cP-ΔCCA-tRNAs were markedly reduced in the *tms5 oshel2-1* mutant. Most of the stalled ribosomes enter the readthrough events, and this portion of the ribosomes participate in the normal translation elongation, termination, and ribosome recycling processes. The dissociated tRNAs replenish the pool of mature tRNAs and alleviate the tRNA repair burden caused by the loss of TMS5, largely restoring male fertility in *tms5 oshel2-1* at restrictive temperatures [103]. Meanwhile, two parallel studies showed that *CSIT1* and *CSIT2*, which encode distinct E3 ubiquitin ligases associated with the RQC pathway, ubiquitinate catalases and reduce ROS accumulation, thereby regulating the critical sterility-inducing temperature (CSIT) in *tms5* lines [104,105]. Furthermore, Zhou et al. demonstrated that tRNA selectivity during RQC regulates the CSIT in TGMS rice [107]. An amino acid substitution (T552I) in the *OsRqc2*, which encodes a rice Rqc2 homolog, increased the CSIT in *tms5* lines through its C-terminal alanine and threonine modification (CATylation) activity. Functional analysis showed that translation-stalling products in plants are subjected to CATylation by OsRqc2. The amino acid residues in the C-terminal tails produced by OsRqc2 are mainly Ala, along with Ser, Thr, Ile, and others. Finally, they found that T552I alted the composition of the C-terminal tails by OsRqc2, resulting in a decreased CATylation rate. Thus, the level of mature tRNA-Ser/Ile is rescued by OsRqc2^T552I^, increasing the CSIT of *tms5* lines [107]. OsRqc1 regulates the CSIT in *tms5* lines by assisting OsVms1 in recruiting 60S ribosomal subunits to generate cP-ΔCCA-tRNAs. Variations in a 6 bp repeat in the 5′ untranslated region (UTR) of OsRqc1 in rice subspecies affect its transcription, thereby altering the CSIT in *tms5* lines. In the *tms5* lines with the *OsRqc1^Hap1^* allele, excess free OsRqc1 competitively binds OsVms1 in the cytoplasm, causing elevated CSIT in *japonica tms5* lines. In *OsRqc1*-knockout *tms5* lines, impaired recruitment of OsVms1 to 60S ribosome-nascent chain complexes (RNCs) partially blocks generation of cP-ΔCCA-tRNAs, which restores mature tRNA levels and increases *tms5* CSIT [108]. Together, these discoveries establish a direct and multifaceted link between RQC pathway components and the molecular mechanisms governing fertility regulation in TGMS rice, highlighting the RQC system as a central hub for integrating translational homeostasis, tRNA metabolism, and ROS balance to control male sterility under temperature stress (Figure 4B).

### 3.8. Identification and Molecular Mechanisms Regulating HGMS in Rice

The pollen wall is a specialized structure essential for pollen development and survival in terrestrial flowering plants, providing physical protection and mediating interactions with the stigma [109]. Very-long-chain fatty acids (VLCFAs) and their derivatives are key components of tryphine, the lipid-rich outer layer of the pollen wall, and play critical roles in enabling pollen to adapt to humidity fluctuations by maintaining hydration and facilitating stigma adhesion [110].

**Figure 5:**
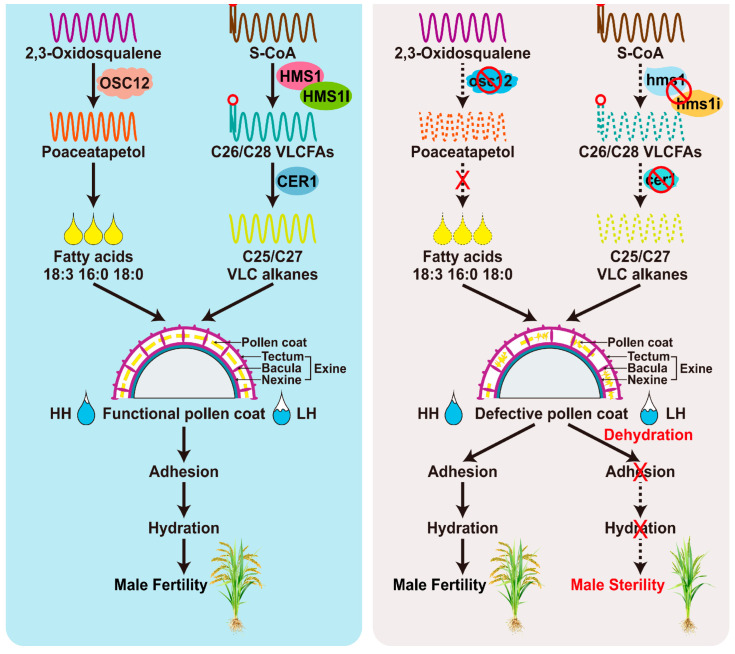
Molecular mechanism of humidity-sensitive genic male sterility (HGMS) associated with tryphine biosynthesis. *OsOSC12*/*OsPTS1* encodes a poaceatapetol synthase to catalyze the cyclization of 2,3-oxidosqualene into poaceatapetol, a bicyclic triterpene that facilitates the deposition of long-chain fatty acids (18:3 16:0 18:0) in the pollen coat via esterification. In the *ososc12* mutant, the levels of long-chain fatty acids, sterols, and several triterpene esters are significantly reduced. These perturbations in pollen coat composition impair pollen adhesion to the stigma and prevent proper hydration, ultimately leading to male sterility under low-humidity conditions. *OsHMS1*, encoding a β-ketoacyl-CoA synthase, interacts with OsHMS1I in the endoplasmic reticulum to catalyze the synthesis of very-long-chain fatty acids (VLCFAs) that are subsequently catalyzed by OsGL1-4/OsCER1 to form very-long-chain alkanes (VLCAs). Mutations of *OsOSC12*, *HMS1* or *HMS1I* result in the formation of disordered tryphine, and deficiencies in VLCFAs, triterpenes, or VLC alkanes lead to rapid pollen dehydration, disrupting stigma adhesion and germination and thus resulting in sterility under low-humidity conditions. HH, high-humidity conditions; LH, low-humidity conditions.

HGMS represents a novel subtype of EGMS, characterized by reduced seed-setting rates under low-humidity conditions and restored normal seed-setting under high-humidity conditions. To date, four HGMS-associated genes have been identified in rice, all of which are linked to pollen wall metabolism, highlighting the central role of pollen coat composition in humidity-dependent fertility regulation. The first reported rice HGMS mutant is *ososc12* [63]. The corresponding gene, *Oxidosqualene Cyclase* (*OsOSC12*)/*Poaceatapetol Synthase* (*OsPTS1*) encodes a bicyclic triterpene synthase that catalyzes the biosynthesis of poaceatapetol, a bicyclic triterpene that facilitates the deposition of long-chain fatty acids in the pollen coat via esterification. In the *ososc12* mutant, the levels of long-chain fatty acids, sterols, and several triterpene esters are significantly reduced, whereas the accumulation of three major phytosterols is increased. These perturbations in pollen coat composition impair pollen adhesion to the stigma and prevent proper hydration, ultimately leading to male sterility under low-humidity conditions. Notably, the fertility of *ososc12* is fully restored under high-humidity conditions, directly demonstrating that the pollen coat is indispensable for supporting pollen adhesion and hydration when environmental humidity is limited [63]. A second HGMS-related gene, *Glossy1-homologous gene* (*OsGL1-4*)/*ECERIFERUM 1* (*OsCER1*), belongs to the Glossy1 family and functions as a key enzyme in the biosynthesis of VLC alkanes [111]. Pollen grains of the *osgl1-4* mutant remain viable but exhibit defective hydration under normal humidity conditions, and this defect is rescued under high humidity. Knockout of *OsGL1-4*/*OsCER1* likely disrupts VLC alkane metabolism and alters the lipid composition of the pollen coat, which in turn compromises pollen adhesion to the stigma, hydration, and subsequent germination [111,112,113]. The third identified gene, *Humidity-sensitive genic Male Sterility 1* (*OsHMS1*), encodes a β-ketoacyl-CoA synthase that plays a key role in the biosynthesis of VLCFAs in rice. OsHMS1 specifically catalyzed the biosynthesis of C26 and C28 VLCFAs, which contribute to the formation of bacula and tryphine, and these components are essential for protecting pollen from dehydration. Under low-humidity conditions, pollen from the *hms1* mutant exhibits impaired adhesion to stigmas and reduced germination on stigmas, whereas these defects can be rescued by increasing environmental humidity. Additionally, HMS1-interacting protein (HMS1I) interacts with HMS1 to coregulate VLCFA biosynthesis and HGMS, further refining the molecular network governing humidity-dependent fertility [114]. Collectively, the mechanisms underlying rice HGMS are primarily centered on the regulation of pollen outer coating components. Deficiencies in triterpenes, VLCFAs, or VLC alkanes lead to rapid pollen dehydration, disrupting stigma adhesion and germination and thus resulting in sterility under low-humidity conditions. In contrast, high humidity (relative humidity exceeds 80%) mitigates these dehydration defects, allowing HGMS mutants to regain fertility (Figure 5). Future exploration of novel components and causal genes involved in pollen coat metabolism will facilitate the identification of additional HGMS regulatory elements, which are critical for developing two-line hybrid systems with enhanced adaptability to drought-prone environments. This HGMS regulatory network, centered on pollen wall lipid metabolism, expands the repertoire of EGMS mechanisms in rice and provides new insights for hybrid rice breeding.

### 3.9. Identification and Molecular Mechanisms Regulating NGMS in Rice

Nitrogen is one of the most indispensable macronutrients for plants, playing a crucial role in modulating multiple plant developmental processes, from vegetative growth to reproductive maturation. During the reproductive stage, spikelets act as the primary sink tissues for nitrogen, thus nitrogen deficiency frequently results in floral abortion and impaired male fertility. NGMS, a newly identified subtype of EGMS, is characterized by male sterility under nitrogen starvation and fertility restoration when exogenous nitrogen is sufficiently supplied. The first characterized rice NGMS mutant is defective in the *electron transfer flavoprotein subunit β* (*etfβ*) mutant. The mutant exhibits complete male sterility under nitrogen-deficient conditions, and this sterility phenotype can be fully rescued by exogenous application of excess exogenous inorganic nitrogen [64]. The *ETFβ* gene encodes a mitochondrion-localized electron transfer flavoprotein subunit β and was involved in the reutilization of branched-chain amino acids through the electron transfer flavoprotein (ETF)/electron transfer flavoprotein quinone oxidoreductase (ETFQO) system [115].

In plants, primary nitrogen assimilation and secondary nitrogen reutilization collectively ensure sufficient nitrogen supply for growth under conditions of sufficient exogenous nitrogen. In contrast, the primary assimilation pathway is severely repressed under nitrogen-deficient conditions, leaving the nitrogen reutilization pathway as the sole source of nitrogen for sink tissues [116]. Nitrogen-containing compounds are hydrolyzed into amino acids, which are further metabolized through the ETF/ETFQO system to release nitrogen for remobilization. Loss of *ETFβ* function disrupts the ETF/ETFQO system, leading to the abnormal accumulation of leucine (Leu), isoleucine (Ile), and their derivatives in the *etfβ* mutant. Under nitrogen-deficient conditions, this accumulation sequesters nitrogen in these uncatabolized amino acids, preventing nitrogen remobilization and reutilization to developing spikelets. The resulting insufficient nitrogen supply to spikelets impairs pollen development, ultimately inducing male sterility. However, under sufficient exogenous nitrogen conditions, the primary nitrogen assimilation pathway is fully active in the *etfβ* mutant. This pathway compensates for the defective nitrogen reutilization system by directly providing adequate nitrogen for spikelet development, thereby restoring male fertility [64]. This discovery of NGMS in the *etfβ* mutant reveals a novel nutrient-dependent regulatory mechanism for EGMS in rice. This finding broadens our understanding of how rice integrates environmental nutrient signals with reproductive development. Practically, this mechanism also holds practical value for developing two-line hybrid rice systems, as it enables precise fertility control through nutrient management, providing a flexible strategy to adapt hybrid seed production to diverse soil nutrient conditions.

## 4. DGMS Germplasm and Molecular Regulation in Rice

In addition to EGMS, another subtype of GMS, namely DGMS, has been identified in rice over the past few decades [117,118]. DGMS is caused by mutations in dominant nuclear genes, and its genetic characteristics are distinctly different from those of CMS and recessive EGMS.

**Figure 6:**
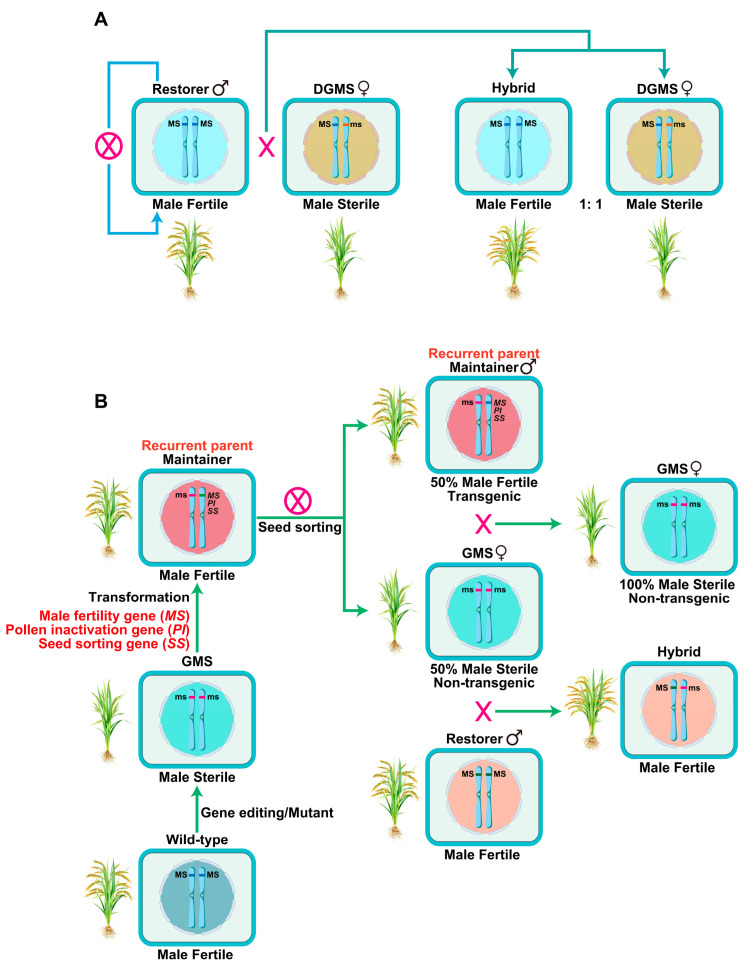
Genetic and mechanistic models of dominant genic male sterility (DGMS) (**A**) and third generation hybrid rice technology (TGHRT) system (**B**) in rice, respectively. (**A**) The DGMS line, which is heterozygous for the dominant gene (MS/ms), is regulated by a single gene or locus and exhibits male sterility under heterozygous genotype. When this DGMS line is crossed with fertile rice plants, its progeny show a 1:1 segregation ratio of male-sterile to fertile individuals. Notably, the male-sterile progeny can be repeatedly propagated and utilized as new DGMS lines for subsequent breeding processes. (**B**) Schematic diagram of the TGHRT system. The genic male sterility (GMS) line is obtained via either mutant screening or CRISPR/Cas9-mediated genetic modification. A single T-DNA cassette containing three functional genes, the male fertility restoration gene (*MS*), pollen inactivation gene (*PI*), and seed sorting gene (*SS*), is transformed into the GMS lines to generate a maintainer line (serving as the recurrent parent). Self-pollination of the maintainer line produces two types of seeds in equal proportion: 50% male-sterile seeds and 50% male-fertile seeds (recurrent parent), which are efficiently separated using the seed-sorting marker. When the male-sterile line is cross-pollinated with the maintainer line (recurrent parent), 100% male-sterile seeds are produced, as transgenic pollen is selectively eliminated by the pollen inactivation gene. Notably, any conventional rice germplasm can be utilized as a restorer line to cross with the GMS line for hybrid rice breeding.

Notably, DGMS lines exhibit a stable sterile phenotype in the heterozygous state. When heterozygous sterile plants are crossed with fertile plants, the resulting progeny exhibit a strict 1:1 segregation ratio of fertile to sterile individuals. Importantly, the fertile progeny do not undergo further fertility segregation in subsequent selfing generations, whereas the sterile progeny can be repeatedly used as sterile lines in breeding practices (Figure 6A). Nevertheless, natural germplasm resources carrying DGMS traits are extremely scarce, which limits their widespread application in hybrid rice breeding [119]. To date, only nine cases of DGMS have been documented in rice. These include naturally occurring or spontaneously discovered lines such as Pingxiang dominant genic male sterile rice [120,121], the low-temperature-sensitive dominant male sterile rice line “8987” [122], Sanming dominant genic male sterile (SDGMS) rice [123], *japonica* dominant genic male-sterile rice W450 [124], and *OsDMS-2* dominant male sterile rice [125]. In addition to these natural variants, three dominant genic male sterile lines, including the Zhe 9248 mutant M1 [126], the Orion mutant 1783, and the Kaybonnet mutant 1789 [127], have been developed via artificial mutagenesis. Notably, the *OsDMS-1* dominant male sterile rice line was obtained through tissue culture-induced variation [128]. Despite the identification of these DGMS lines, the regulatory genes and their underlying molecular mechanisms remain unclear in most cases.

A breakthrough in DGMS research was achieved with the first successful cloning of the causal gene underlying DGMS in SDGMS rice. Unlike EGMS lines, SDGMS lines exhibit complete and stable male sterility across different day lengths and temperatures. This study revealed that the spontaneous insertion of a 1978 bp long terminal repeat (LTR) retrotransposon into the promoter region of the *SDGMS* gene drives its specific overexpression in the anther tapetum. *SDGMS* encodes a ribosome-inactivating protein with N-glycosidase activity, and its tapetum-specific activation triggers abnormal PCD of tapetal cells, ultimately leading to dominant male sterility. Furthermore, the activation of *SDGMS* induces transcriptional reprogramming of biotic stress-responsive genes, initiating a hypersensitive response reaction in anthers. This immune-related response further contributes to the sterility phenotype [129]. Recently, another regulatory mechanism of SDGMS was identified, involving an epigenetic allele of *Sanming dominant male sterility* (*SMS*). In the male-sterile 93-11 near-isogenic line (NIL), the *SMS* locus is heterozygous, harboring an epi-allele identical to that in 93-11 and an epi-allele identical to that in rice cultivar Nipponbare, designated as *SMS_9_* and *SMS_N_*, respectively. In this 93-11 NIL, *SMS_9_* is transcriptionally silent and hyper-methylated, whereas *SMS_N_* is expressed and hypo-methylated. Functional validation showed that overexpression of *SMS_N_* led to male sterility, while mutations in *SMS* rescue the sterility of the 93-11 NIL. These results demonstrate that the reduced methylation and enhanced expression of the *SMS_N_* epi-allele in the 93-11 NIL are responsible for conferring dominant male sterility, highlighting epigenetic regulation as a novel mechanism underlying DGMS in rice [130].

## 5. Development of Biotechnology-Based TGHRT

The TGHRT is a transgenic-based system designed for the propagation and utilization of stable recessive GMS lines, which marks a significant breakthrough in enhancing the efficiency of hybrid seed production [7,131,132]. This system is derived from the seed production technology (SPT) initially developed by researchers at Dupont Pioneer for maize. The maize SPT system was established by introducing a transgene cassette into the male-sterile mutant *ms45*. This cassette comprises three key elements: the wild-type fertility restorer gene *MS45*, the maize α-amylase gene *ZmAA1* (serving as a pollen-inactivating gene), and the red fluorescent protein gene *DsRed2* (acting as a seed-specific marker) [133]. The functional specialization of these transgenes is critical to the operation of SPT: the *ZmAA1* gene disrupts starch biosynthesis in the pollen grains, thereby inactivating transgenic pollen; the *DsRed2* gene drives the production of red fluorescence in transgenic seeds, facilitating both visual and instrumental sorting; and the *MS45* restores fertility to the *ms45* mutant, enabling self-pollination of the transgenic line. Transgenic maize plants carrying a single copy of this SPT cassette produce two distinct pollen types in a 1:1 ratio: 50% are non-transgenic and fertile, while the remaining 50% are transgenic and sterile. Notably, the maize SPT system has been commercially applied in hybrid maize breeding and production in the United States since 2012 [133].

To adapt the SPT framework for commercial hybrid rice breeding, researchers screened a mutant library of the *indica* rice variety Huanghuazhan (HHZ) and identified the recessive GMS mutant *osnp1*. The mutant exhibits normal vegetative growth, a high stigma exsertion rate, a high outcrossing rate, and stable male sterility across diverse environmental conditions. Molecular characterization revealed that *OsNP1* encodes a glucose-methanol-choline oxidoreductase, with its expression specifically localized to the anther tapetum and microspores, which was consistent with its role in pollen development [134]. To develop the corresponding maintainer line, the research team constructed a single T-DNA cassette containing three key functional elements: the wild-type *OsNP1* gene (for restoring fertility to *osnp1*), the pollen-inactivating gene *ZmAA1*, and the red fluorescent seed-marker gene DsRed2. This cassette was then introduced into the *osnp1* mutant, resulting in the generation of the maintainer line Zhen18B, which harbors a single copy of the transgene. When Zhen18B undergoes self-pollination, it produces seeds in a strict 1:1 ratio: non-transgenic male-sterile seeds (designated as Zhen18A, the sterile line for hybrid production) and transgenic fertile seeds (Zhen18B, the maintainer line). Critically, these two seed types can be efficiently separated using a fluorescence-activated sorter, ensuring the high-purity recovery of Zhen18A. Outcrossing Zhen18A with Zhen18B yields large quantities of high-purity non-transgenic male-sterile seeds, laying a solid foundation for large-scale hybrid seed production (Figure 6B). To evaluate the breeding potential of Zhen18A, Zhen18A plants were crossed with approximately 120 diverse rice germplasms. Notably, approximately 85% of the resulting F_1_ progeny exhibited higher per-plant yield compared to their respective parental lines, confirming the system’s effectiveness in developing high-yield hybrid varieties [134]. This rice-adapted SPT system was later designated TGHRT, highlighting its significance as a scalable and efficient tool for hybrid rice breeding that complements traditional breeding systems [7,131,132].

## 6. Future Prospects and Conclusions

Over the past five decades, hybrid rice technology has emerged as a transformative force in agricultural production, driving a substantial increase in grain yield and thus making an irreplaceable contribution to safeguarding China’s food supply security [3,4,5]. The development of the first-generation three-line hybrid rice system based on CMS in the 1970s and the second-generation two-line hybrid rice system based on EGMS in the 1980s has significantly boosted rice yields and strengthened food security in China [12,13,135]. In recent years, with the development of genetic engineering and molecular biology, third-generation hybrid rice has opened up a new avenue for the evolution of hybrid rice technology [7,134]. Each of these systems possesses unique advantages while facing distinct challenges.

### 6.1. Application and Limitation of CMS in Rice

In the early 1970s, three-line hybrid rice varieties with significant potential for large-scale agricultural application were successfully developed, marking a pivotal breakthrough in hybrid rice technology [19]. Following the discovery of the CMS-WA system, a series of elite CMS lines were successfully developed, with representative varieties including Zhenshan 97A, V20A, and Jing 23A. Meanwhile, to match these CMS lines, corresponding restorer lines (e.g., Taiyin No. 1, IR8, IR24, IR26, Milyang 23, and Milyang 46) were screened and identified through systematic test-crossing experiments [19]. To date, many of these early-developed sterile lines and restorer lines remain core germplasm resources, providing critical genetic material support for the breeding of new male sterile lines and restorer lines in hybrid rice research. In the 1970s, China achieved a major breakthrough in the commercialization of three-line hybrid rice: the first commercial hybrid rice variety, Nanyou 2, was released, followed by the widespread promotion of Weiyou 6. Field yield trials and large-scale production practices demonstrated that these pioneering three-line hybrid rice varieties exhibited an approximate 20% yield advantage over elite conventional rice varieties. This significant yield increase laid a solid foundation for the rapid large-scale commercial production of three-line hybrid rice in China, representing a key milestone in the application of hybrid rice technology to address global food security challenges [19]. To date, the sown area of the first-generation hybrid rice based on three-line system still accounts for more than half of the total sown area of hybrid rice worldwide, underscoring its enduring significance [20,61].

Globally, three-line hybrid rice has been widely adopted in over 60 countries, including major rice-producing regions such as China, Vietnam, Indonesia, the Philippines, and the United States [136]. As a result, its extensive implementation has empowered rice producers to enhance yields and stabilize production, making substantial contributions to global food security. However, the further advancement of three-line hybrid rice breeding is constrained by two critical bottlenecks: the scarcity of restorer lines and insufficient genetic diversity between CMS lines and restorer lines, which limits the development of novel heterotic combinations. Furthermore, the induction of CMS is dependent on the presence of nuclear-encoded *Rf* genes under cytoplasmic sterile backgrounds. This inherent genetic constraint means that not all rice germplasm can be developed into CMS lines.

Practical breeding data indicate that the conversion rate of *indica* rice varieties into CMS lines is merely 0.1%. Even among the few successfully converted lines, only approximately 5% can be practically applied in hybrid seed production, primarily due to agronomic defects such as poor floral traits or unstable sterility. This low availability of qualified CMS lines significantly reduces the efficiency of selecting superior hybrid combinations. Another critical challenge in CMS line application is fertility instability at high temperatures. When some CMS lines are exposed to sustained high temperatures during the flowering period, a certain proportion of sterile plants will regain partial fertility. This phenomenon directly impairs the purity of hybrid seeds and increases seed production costs. Collectively, these limitations have hindered the further promotion and application of CMS in exploiting rice heterosis [20].

### 6.2. Application and Limitation of EGMS in Rice

NK58S, the first EGMS rice, exhibits male sterility under LD conditions while restoring male fertility under SD conditions. This PGMS line was first discovered by Professor Mingsong Shi in 1973 [137], and its discovery provided a novel genetic resource for the development of two-line hybrid rice systems [138]. The availability of EGMS germplasms has greatly facilitated the development of two-line hybrid rice, which possesses distinct advantages over the traditional three-line hybrid rice system. First, EGMS rice lines can self-propagate under permissive environmental conditions and produce hybrid seeds under restrictive conditions, thereby eliminating the need for dedicated maintainer lines [139]. This unique characteristic significantly reduces labor input, time consumption, and resource costs in the hybrid rice production. Second, the two-line hybrid system is governed by nuclear genes, enabling EGMS lines to cross with a broad spectrum of conventional rice varieties. Such flexibility in parental line matching not only expands the germplasm pool for restorer line selection but also enhances the efficiency of heterosis utilization. Owing to these prominent merits, the development of two-line hybrid rice has long remained a research focus for scientists and breeders. Currently, breeders primarily rely on P/TGMS lines for two-line hybrid breeding, with TGMS lines being the most widely utilized. Notably, two-line hybrid rice derived from P/TGMS lines were commercially released in 1996, exhibiting high yield potential and improved grain quality [139,140]. However, their large-scale application is still constrained by several bottlenecks, including the limited diversity of EGMS germplasm resources, the lack of genetically pure PGMS lines, and the complicated regulatory mechanism of the CSIT in TGMS lines.

Although numerous EGMS loci have been identified in rice, the majority remain uncloned, and few corresponding germplasms of the characterized genes are applicable to hybrid seed production due to the adverse side effects exerted by many of these genes [141]. There are still numerous challenges in cloning these genes and investigating their molecular mechanisms, including difficulties in phenotype identification and the complex genetic basis of EGMS lines. Due to frequent fluctuations in environmental factors, the fertility phenotype of EGMS lines often varies, which may interfere with subsequent gene mapping and cloning. Therefore, precise characterization of the anther development stage of each EGMS line, selection of optimal planting windows, and establishment of a robust platform for accurate fertility phenotyping are of great significance for advancing EGMS gene cloning, dissecting their molecular mechanism, and promoting their practical application in hybrid rice breeding. Moreover, a major shift from PGMS-based to TGMS-based two-line hybrid rice occurred in China’s rice production between 1993 and 2012. In the early stages of two-line hybrid rice breeding, the P/TGMS genes *pms1* and *pms3* (also known as *p/tms12-1*) were initially employed. However, they have since been replaced by the TGMS gene *tms5* over the past few decades. By 2012, TGMS lines containing *tms5* had significantly advanced two-line hybrid rice breeding, with *tms5*-containing hybrids occupying more than 95% of the planting area in two-line hybrid rice production [142]. This excessive reliance on a single gene is unfavorable for fully unlocking the potential of two-line hybrid breeding, as it limits genetic diversity and increases vulnerability to environmental fluctuations [142,143]. To promote the application of EGMS genes in hybrid rice breeding, several key steps should be implemented. First, efforts should be made to clone as many EGMS genes as possible and elucidate their underlying regulatory mechanisms. Subsequently, EGMS mutant libraries could be constructed via mutagenesis and gene editing techniques, which could provide both technical support and germplasm resources for the subsequent evaluation of application potential in hybrid rice breeding.

The safety of F_1_ hybrid seed production represents the most critical challenge in two-line hybrid rice breeding. Considering the global harvested area of rice, climate models predict that by 2030, 16% of rice-growing regions will be exposed to temperatures exceeding the critical threshold for at least 5 days during the reproductive period, with this proportion increasing non-linearly to 27% by 2050 [144]. Against such a backdrop, EGMS lines are prone to partial fertility restoration and subsequent self-pollination during hybrid seed production due to abnormal fluctuations in ambient conditions, which directly impairs the safety and genetic purity of F_1_ hybrid seeds. Conceptually, PGMS lines were assumed to have more stable sterility–fertility transition under environmental fluctuations than TGMS lines. This is because the photoperiod remains relatively consistent in specific geographical regions, in sharp contrast to the highly erratic nature of temperature fluctuations [62]. However, no purely PGMS gene has been identified to date. The fertility of PGMS lines is not only regulated by the photoperiod but also influenced by temperature. Furthermore, when introduced into different genetic backgrounds, PGMS genes may even confer TGMS traits, further complicating their application [62,65]. For example, the PGMS line NK58S showed temperature-dominated sterility after its photoperiod-sensitive gene was introduced into *indica* rice PA64S [13]. Similarly, the *csa* gene exhibited rPGMS in *japonica* rice ‘9522’, but both rPGMS and rTGMS traits in *japonica* rice ‘Kongyu 131’ [79,145]. With the increasing accumulation of gene expression profile data, it has become feasible to identify genes that play essential roles in pollen development, either exhibiting clear photoperiod dependence or remaining unaffected by genetic background or temperature fluctuations. Given the growing complexity of environmental factors, developing feasible strategies to address the aforementioned challenges in two-line hybrid rice breeding is imperative. First, pyramiding multiple P/TGMS loci through conventional breeding approaches or advanced gene editing technologies facilitates the generation of EGMS lines with enhanced fertility stability. This advantage is attributed to the synergistic effects of stacked loci, which can effectively buffer the disruptive impacts of environmental fluctuations on the sterility–fertility conversion process. Second, the development of novel sterile lines insensitive to both genetic background and temperature fluctuations provides a direct solution to overcome the limitations imposed by background-dependent trait variation and erratic temperature shifts. By decoupling sterility regulation from external genetic and environmental interferences, such lines can maintain consistent sterility under diverse field conditions. When implemented individually or in combination, these two strategies not only enhance the environmental adaptability of EGMS lines across varied agroecological zones but also improve the practical utility of characterized EGMS genes, thereby promoting the scalability and sustainability of two-line hybrid rice production systems.

CSIT is defined as the threshold temperature at which TGMS lines transition from a fertile to a completely sterile state, and it exhibits substantial variation among *tms5*-containing lines [146,147,148,149]. Notably, the *tms5* lines of *japonica* rice varieties with relatively high CSIT values (ranging from 28 °C to 32 °C) are particularly susceptible to hybrid seed production failure when exposed to abnormally low temperatures, resulting in significant economic losses [108]. Male sterile lines with high CSIT and short low-temperature tolerance duration are more likely to encounter temperatures below their CSIT during seed production. This can lead to hybrid seed production failure, as the male sterile lines regain selfing fertility under such temperature conditions [150]. Historically, large-scale two-line hybrid rice seed production failures have occurred multiple times due to sterility fluctuation induced by high CSIT and short low-temperature tolerance duration of male sterile lines. For instance, in Hunan Province in 1993, 1996, and 1999, low-temperature events during the fertility-sensitive period of male sterile lines resulted in widespread seed production failure, causing extremely heavy economic losses [150]. Therefore, developing TGMS lines with a moderately low CSIT has emerged as a critical priority for ensuring the safety of two-line hybrid rice seed production. Based on breeders’ practical experience, a consensus has been reached that the optimal CSIT range for TGMS lines is 21–24 °C [151]. A CSIT exceeding 24 °C compromises the safety of hybrid seed production, whereas a CSIT below 21 °C hinders plant self-pollination. Even when the fertility-sensitive period of these male sterile lines is scheduled for July to August, the warmest months of the year, it remains difficult to avoid episodes of temperature dropping below 23.5 °C, thereby posing potential safety risks to hybrid seed production. A typical example is the two-line hybrid rice seed production failures covering approximately 6700 hm^2^ in Yancheng, Jiangsu Province, in 2009 and 2011. These incidents caused direct economic losses exceeding 100 million yuan. More notably, they disrupted the safe supply of hybrid rice seeds in the subsequent year, leading to incalculable indirect losses [150].

Therefore, the core technical approach to mitigating the risks in two-line hybrid rice seed production lies in breeding practical TGMS lines characterized by a low CSIT and an extended duration of low-temperature tolerance. If the CSIT can be lowered to 22–22.5 °C, and the low-temperature tolerance duration prolonged to 6 days, the risks associated with mid-summer seed production in the Yangtze River Basin will be significantly mitigated [150,152]. The deployment of TGMS lines with superior low CSIT and prolonged low-temperature tolerance not only enhances the safety and stability of seed production, but also expands the selection range of seed production bases, thereby facilitating the identification of high-yielding bases with reliable safety profiles [150,152]. Recent studies have demonstrated that CSIT1/OsLtn1, CSIT2/OsHel2, OsVms1, OsRqc2, and OsRqc1, which are associated with the RQC pathway, cooperate with TMS5 to regulate CSIT in *tms5* lines [103,104,105,106,107,108]. These findings may facilitate the molecular breeding of *tms5*-based two-line hybrid rice lines with tailored CSIT values, enabling adaptation to geographically diverse agroecological regions [108]. However, significant variations in CSIT have been observed among different TGMS lines containing the *tms5* locus but with distinct genetic backgrounds [149]. Furthermore, the CSIT of the EGMS lines often increases after several generations of propagation, ultimately rendering them unsuitable for hybrid rice breeding [62,132]. The CSIT trait is regulated by multiple minor-effect genes; consequently, heterozygosity at these minor loci is considered the primary driver of CSIT drift in TGMS lines carrying the same *tms5* mutation [153]. Therefore, cloning these minor-effect genes will be crucial for reducing the CSIT of *tms5*-containing TGMS lines in future research.

### 6.3. Application and Limitation of TGHRT in Rice

Male reproductive development in plants is a sophisticated biological process consisting of three sequential stages: stamen formation accompanied by the differentiation of anther tissues, where microspores/pollen grains are produced, followed by anther dehiscence, and ultimately pollination. The normal progression of these stages depends on the coordinated interaction between sporophytic and gametophytic genes [77]. As a model monocot crop, rice follows this conserved biological pattern for stamen development and pollen formation, albeit regulated by a unique set of GMS genes. As reported, over 50 GMS genes have been identified in rice, highlighting the genetic complexity underlying the regulation of male fertility [154,155,156,157,158,159]. To exploit the utility of these GMS genes, GMS lines can be developed in rice germplasm via CRISPR/Cas9-mediated gene editing.

A prominent application of this strategy is the development of TGHRT based on the *OsCYP703A3* gene [160]. *OsCYP703A3* encodes a cytochrome P450 hydroxylase, an enzyme essential for pollen wall formation in rice. This gene exhibits anther-specific expression, with its transcripts detectable from the tetrad stage to the binuclear microspore stage of anther development [161,162]. To generate a GMS line, *OsCYP703A3* was knocked out in the elite rice variety 9311 using CRISPR/Cas9 technology, yielding in the *9311^03a3^* mutant. Subsequently, a maintainer line (9311-3B) was constructed by transforming the *9311^03a3^* mutant with a transgene cassette containing the wild-type *OsCYP703A3* gene, the pollen-inactivating gene *orfH79*, and the seed-sorting marker gene *DsRed2*. Self-pollination of 9311-3B produced a male-sterile line (9311-3A) without the transgenic component and the maintainer line 9311-3B carrying the transgenic component in a 1:1 ratio. Based on this TGHRT system, several high-performance hybrid combinations have been developed [160]. Among these, Sanyou No.1, a hybrid rice derived from 9311-3A, has broken China’s existing rice yield record, achieving a double-cropping yield exceeding 1500 kg per mu (667 m^2^). This achievement represents a historic breakthrough in rice production and underscores the substantial practical value of TGHRT [160]. Collectively, these preliminary results further demonstrate the broad application prospects of TGHRT in advancing hybrid rice breeding.

To date, promising practical application outcomes of TGHRT have only been reported for two systems: the *OsNP1*-based Zhen18B system and *OsCYP703A3*-based 9311-3B system [160]. Notably, both of these previously reported female-sterility maintainer systems are technically sound and mature, with robust performance in laboratory validation and field trials. Thus, these systems provide reliable technical support for large-scale commercial breeding and hold significant practical utility in hybrid rice production [163,164]. Such progress remains far from meeting the demand for hybrid rice varieties capable of adapting to diverse geographical regions and environmental conditions. Although a large number of GMS genes have been cloned in rice, not all exhibit anther-specific expression. Instead, some are also transcribed in other vegetative and/or reproductive tissues, indicating broader spatial expression profiles [132,154,165]. Such genes likely perform functions beyond regulating pollen development. Consequently, mutations in these genes may induce adverse pleiotropic effects on the growth and development of non-target tissues, posing a significant constraint for their application in GMS line development.

In addition, in compliance with China’s current regulatory framework for genetically modified organisms (GMOs), a newly constructed de novo maintainer line must complete three sequential testing phases: intermediate testing, environmental release testing, and production testing prior to the issuance of a safety certificate [166,167]. This mandatory testing requires extensive experimental validation, substantial financial investment, and a minimum timeframe of four years from the initiation of testing to eligibility for safety certificate application. Despite these regulatory hurdles, it is anticipated that with the widespread popularization and application of related technologies in the future, the third-generation hybrid rice will open up a new avenue for boosting rice production in China.

## Data Availability

No new data were created or analyzed in this study. Data sharing is not applicable to this article.
